# Assessing scientists for hiring, promotion, and tenure

**DOI:** 10.1371/journal.pbio.2004089

**Published:** 2018-03-29

**Authors:** David Moher, Florian Naudet, Ioana A. Cristea, Frank Miedema, John P. A. Ioannidis, Steven N. Goodman

**Affiliations:** 1 Centre for Journalology, Clinical Epidemiology Program, Ottawa Hospital Research Institute, Ottawa, Canada; 2 Meta-Research Innovation Center at Stanford (METRICS), Stanford University, Stanford, California, United States of America; 3 INSERM CIC-P 1414, Clinical Investigation Center, CHU Rennes, Rennes 1 University, Rennes, France; 4 Department of Clinical Psychology and Psychotherapy, Babeş-Bolyai University, Cluj-Napoca, Romania; 5 Executive Board, UMC Utrecht, Utrecht University, Utrecht, the Netherlands; 6 Department of Medicine, Stanford University, Stanford, California, United States of America; 7 Department of Health Research and Policy, Stanford University, Stanford, California, United States of America; 8 Department of Biomedical Data Science, Stanford University, Stanford, California, United States of America; 9 Department of Statistics, Stanford University, Stanford, California, United States of America

## Abstract

Assessment of researchers is necessary for decisions of hiring, promotion, and tenure. A burgeoning number of scientific leaders believe the current system of faculty incentives and rewards is misaligned with the needs of society and disconnected from the evidence about the causes of the reproducibility crisis and suboptimal quality of the scientific publication record. To address this issue, particularly for the clinical and life sciences, we convened a 22-member expert panel workshop in Washington, DC, in January 2017. Twenty-two academic leaders, funders, and scientists participated in the meeting. As background for the meeting, we completed a selective literature review of 22 key documents critiquing the current incentive system. From each document, we extracted how the authors perceived the problems of assessing science and scientists, the unintended consequences of maintaining the status quo for assessing scientists, and details of their proposed solutions. The resulting table was used as a seed for participant discussion. This resulted in six principles for assessing scientists and associated research and policy implications. We hope the content of this paper will serve as a basis for establishing best practices and redesigning the current approaches to assessing scientists by the many players involved in that process.

## Introduction

Assessing researchers is a focal point of decisions about their hiring, promotion, and tenure. Building, writing, presenting, evaluating, prioritising, and selecting curriculum vitae (CVs) is a prolific and often time-consuming industry for grant applicants, faculty candidates, and assessment committees. Institutions need to make decisions in a constrained environment (e.g., limited time and budgets). Many assessment efforts assess primarily what is easily determined, such as the number and amount of funded grants and the number and citations of published papers. Even for readily measurable aspects, though, the criteria used for assessment and decisions vary across settings and institutions and are not necessarily applied consistently, even within the same institution. Moreover, several institutions use metrics that are well known to be problematic [1]. For example, there is a large literature on the problems with and alternatives to the journal impact factor (JIF) for appraising citation impact. Many institutions still use it to assess faculty through the quality of the literature they publish in, or even to determine monetary rewards [2].

That faculty hiring and advancement at top institutions requires papers published in journals with the highest JIF (e.g., *Nature*, *Science*, *Cell*) is more than just a myth circulating among postdoctoral students [3–6]. Emphasis on JIF does not make sense when only 10%–20% of the papers published in a journal are responsible for 80%–90% of a journal’s impact factor [7,8]. More importantly, other aspects of research impact and quality, for which automated indices are not available, are ignored. For example, faculty practices that make a university and its research more open and available through data sharing or education could feed into researcher assessments [9,10].

Few assessments of scientists focus on the use of good or bad research practices, nor do currently used measures tell us much about what researchers contribute to society—the ultimate goal of most applied research. In applied and life sciences, the reproducibility of findings by others or the productivity of a research finding is rarely systematically evaluated, in spite of documented problems with the published scientific record [11] and reproducibility across all scientific domains [12–13]. This is compounded by incomplete reporting and suboptimal transparency [14]. Too much research goes unpublished or is unavailable to interested parties [15].

Using more appropriate incentives and rewards may help improve clinical and life sciences and their impact at all levels, including their societal value. We set out to ascertain what has been proposed to improve the evaluation of ‘life and clinical’ research scientists, how a broad spectrum of stakeholders view the strengths and weaknesses of these proposals, and what new ways of assessing scientists should be considered.

## Methods

To help address this goal, we convened a 1-day expert panel workshop in Washington, DC, in January 2017. Pre-existing commentaries and proposals to assess scientists were identified with snowball techniques [16] (i.e., an iterative process of selecting articles; the process is often started with a small number of articles that meet inclusion criteria; see below) to examine the literature to ascertain what other groups are writing, proposing, or implementing in terms of how to assess scientists. We also searched the Meta-Research Innovation Center at Stanford (METRICS) research digest and reached out to content experts. Formal searching proved difficult (e.g., exp*Reward/(7408); reward*.ti,kw (9708), incentiv*.ti,kw. (5078)) and resulted in a very large number of records with low sensitivity and recall. We did not set out to conduct a formal systematic review of every article on the topic.

Broad criteria were used for article inclusion (the article focus had to be either bibliometrics, research evaluation, and/or management of scientists and it had to be reported in English). Two of us selected the potential papers and at least three of us reviewed and discussed each selection for its inclusion. From each included article we extracted the following information: authors, name of article/report and its geographic location, the authors’ stated perspective of the problem assessing research and scientists, the authors’ description of the unintended consequences of maintaining the current assessment scheme, the article’s proposed solutions, and our interpretation of the potential limitations of the proposal. The resulting table (early version of Table 1) along with a few specific publications was shared with the participants in advance of the meeting in the hopes that it would stimulate thinking on the topic and be a reference source for discussions.

**Table 1:** A list of sources examining the problems, potential unanticipated consequences, proposed solutions, and potential limitations when assessing science and scientists.

Document name	Geographic region	The stated perspective of the problems assessing science and scientists	Unintended consequences of maintaining the current assessment scheme	Proposed solutions for assessing scientists	Potential limitations of proposal
**Larger group proposals**					
ACUMEN (2014) [17]	Europe	The ACUMEN consortium was focused on ‘understanding the ways in which researchers are evaluated by their peers and by institutions, and at assessing how the science system can be improved and enhanced’. They noted that, ‘Currently, there is a discrepancy between the criteria used in performance assessment and the broader social and economic function of scientific and scholarly research’.	The report points to five problems: 1. ‘Evaluation criteria are still dominated by mono-disciplinary measures, which reflect an important but limited number of dimensions of the quality and relevance of scientific and scholarly work’; 2. ‘Increase the pressure on the existing forms of quality control and evaluation’; 3. ‘The evaluation system has not been able to keep up sufficiently with the transformations in the way researchers create knowledge and communicate their research to colleagues and the public at large’; 4. The bibliometrics in current use ‘do not produce viable results at the level of the individual researcher’; 5. The current ‘scientific and scholarly system has a gender bias’.	The consortium has developed criteria and guidelines for GEP and has designed a prototype for a web-based ACUMEN performance portfolio. The GEP focuses on three indicators for academic assessment: (1) expertise, (2) outputs, and (3) impacts. The performance portfolio is divided into four parts: (1) narrative and academic age calculation, (2) expertise, (3) output, and (4) influence.	The ACUMEN portfolio stresses the inclusion of an evidence-based narrative, in which researchers tell ‘their story’. The risk is that researchers might be nudged to mold or distort their work and achievements to make them fit in a compelling narrative. Moreover, the already prevalent self-marketing techniques (i.e., how to best ‘sell’ yourself) risk becoming even more pervasive and interfering with the quality of research. Some valuable researchers might not have a coherent story to tell nor be adept narrators.
Amsterdam Call for Action on Open Science (2016) [18]	Europe	Participants at the Open Science—From Vision to Action—Conference noted that ‘Open science presents the opportunity to radically change the way we evaluate, reward, and incentivise science. Its goal is to accelerate scientific progress and enhance the impact of science for the benefit of society’.	Conference participants argue that maintaining the current scheme will continue to create a climate of disconnect between old-world bibliometrics and newer approaches, such as a commitment to open science—‘This emphasis does not correspond with our goals to achieve societal impact alongside scientific impact’.	The conference participants’ vision is for ‘New assessment, reward and evaluation systems. New systems that really deal with the core of knowledge creation and account for the impact of scientific research on science and society at large, including the economy, and incentivise citizen science’. To reach this goal, the Call for Action recommends action in four areas: (1) complete OA, (2) data sharing, (3) new ways of assessing scientists, and (4) introducing evidence to inform best practices for the first three themes. Twelve action items are proposed: to (1) change assessment, evaluation, and reward systems in science; (2) facilitate text and data mining of content; (3) improve insight into IPR and issues such as privacy; (4) create transparency on the costs and conditions of academic communication; (5) introduce FAIR and secure data principles; (6) set up common e-infrastructures; (7) adopt OA principles; (8) stimulate new publishing models for knowledge transfer; (9) stimulate evidence-based research on innovations in open science; (10) develop, implement, monitor, and refine OA plans; (11) involve researchers and new users in open science; and (12) encourage stakeholders to share expertise and information on open science.	Although the Amsterdam Call provides several actionable steps stakeholders can take, some of the actions face barriers to implementation. For example, to change assessment, evaluation, and reward schemes, one action is to sign DORA, although few organizations have done so. Similarly, even if hiring, promotion, and tenure committees wanted to move away from focusing solely on JIFs, it is unclear whether they could do so without the broader research institution agreeing to such modifications in assessing scientists.
DORA (2012) [19]	International	At the 2012 annual meeting of the American Society for Cell Biology, a group of publishers and editors noted ‘a pressing need to improve the ways in which the output of scientific research is evaluated by funding agencies, academic institutions, and other parties’. In response, the San Francisco DORA was produced.	DORA points to the critical problems with using JIF as a measure of a scientist’s worth: ‘the Journal Impact Factor has a number of well-documented deficiencies as a tool for research assessment’.	DORA has one general recommendation—do not use journal-based metrics, such as JIFs, as surrogate measures of the quality of individual research articles to assess an individual scientist’s contributions in hiring, promotion, or funding decisions and 17 specific recommendations, for researchers: (1) focus on content; (2) cite primary literature; (3) use a range of metrics to show the impact of your work; (4) change the culture; funders: (5) state that scientific content of a paper, not the JIF of the journal in which it was published, is what matters; (6) consider value from all outputs and outcomes generated by research; research institutions: (7) when hiring and promoting, state that scientific content of a paper, not the JIF of the journal in which it was published, is what matters; (8) consider value from all outputs and outcomes generated by research; publishers: (9) cease to promote journals by impact factor; (10) provide an array of metrics; (11) focus on article-level metrics; (12) identify different author contributions, open the bibliometric citation data; (13) encourage primary literature citations; and organizations that supply metrics: (14) be transparent; (15) provide access to data; (16) discourage data manipulation; and (17) provide different metrics for primary literature and reviews.	There is a focus on citing primary research. Within medicine, citing systematic reviews is often preferred. A clarification on this point would be useful. DORA is silent on how stakeholders should optimally implement their recommendations. Similarly, DORA does not provide guidance on whether (and how) hiring, promotion, and tenure committees should monitor adherence to and implementation of their recommendations.
The Leiden Manifesto (2015) [20]	International	At the 2014 International Conference on Science and Technology Indicators in Leiden, a group of scientometricians met to discuss how data are being used to govern science, including evaluating scientists. The manifesto authors observed that ‘research evaluations that were once bespoke and performed by peers are now routine and reliant on metrics’.	‘The problem is that evaluation is now led by the data rather than by judgement. Metrics have proliferated: usually well intentioned, not always well informed, often ill applied’.	The Leiden Manifesto proposes 10 best practices: (1) quantitative evaluation should support qualitative, expert assessment; (2) measure performance against the research missions of the institution, group, or researcher; (3) protect excellence in locally relevant research; (4) keep data collection and analytical processes open, transparent, and simple; (5) allow those evaluated to verify data and analysis; (6) account for variation by field in publication and citation practices; (7) base assessment of individual researchers on a qualitative judgment of their portfolio; (8) avoid misplaced concreteness and false precision; (9) recognise the systemic effects of assessment and indicators; and (10) scrutinise indicators regularly and update them. Abiding by these 10 principles, research evaluation can play an important part in the development of science and its interactions with society.	The focus of the Leiden Manifesto is on research metrics. Beyond the focus on metrics, it is unclear what the broader scientific community thinks of these principles. Similarly, it is not clear how best promotion and tenure committees might implement them. For example, while allowing scientists to review and verify their evaluation data (principle 5), it is less clear as to how this could be easily monitored. For example, should there be audit and feedback about each principle?
Wilsdon (The Metric Tide) (2015) [21]	United Kingdom	The report was initiated to evaluate the role of metrics in research assessment and management as part of the UK’s REF. The report takes a ‘deeper look at potential uses and limitations of research metrics and indicators. It has explored the use of metrics across different disciplines, and assessed their potential contribution to the development of research excellence and impact’.	The report raises concerns ‘that some quantitative indicators can be gamed, or can lead to unintended consequences; journal impact factors and citation counts are two prominent examples’.	The report proposes five attributes to improve the assessment of researchers: (1) robustness, (2) humility, (3) transparency, (4) diversity, and (5) reflexivity. The report also makes 20 recommendations dealing with a broad spectrum of issues related to research assessment for stakeholders to consider: (1) the research community should develop a more sophisticated and nuanced approach to the contribution and limitations of quantitative indicators; (2) at an institutional level, higher education institution leaders should develop a clear statement of principles on their approach to research management and assessment, including the role of quantitative indicators; (3) research managers and administrators should champion these principles and the use of responsible metrics within their institutions; (4) human resources managers and recruitment or promotion panels in higher education institutions should be explicit about the criteria used for academic appointment and promotion decisions; (5) individual researchers should be mindful of the limitations of particular indicators; (6) research funders should develop their own context-specific principles for the use of quantitative indicators in research assessment and management; (7) data providers, analysts, and producers of university rankings and league tables should strive for greater transparency and interoperability between different measurement systems; (8) publishers should reduce emphasis on JIFs as a promotional tool, and only use them in the context of a variety of journal-based metrics that provide a richer view of performance; (9) there is a need for greater transparency and openness in research data infrastructure; (10) a set of principles should be developed for technologies, practices, and cultures that can support open, trustworthy research information management; (11) the UK research system should take full advantage of ORCID as its preferred system of unique identifiers. ORCID IDs should be mandatory for all researchers in the next REF; (12) identifiers are also needed for institutions, and the most likely candidate for a global solution is the ISNI, which already has good coverage of publishers, funders, and research organizations; (13) publishers should mandate ORCID IDs and ISNIs and funder grant references for article submission and retain this metadata throughout the publication life cycle; (14) the use of DOIs should be extended to cover all research outputs; (15) further investment in research information infrastructure is required; (16) HEFCE, funders, HEIs, and Jisc should explore how to leverage data held in existing platforms to support the REF process, and vice versa; (17) BIS should identify ways of linking data gathered from research-related platforms (including Gateway to Research, Researchfish, and the REF) more directly to policy processes in BIS and other departments; in assessing outputs, we recommend that quantitative data—particularly around published outputs—continue to have a place in informing peer-review judgements of research quality; in assessing impact, we recommend that HEFCE and the UK HE funding bodies build on the analysis of the impact case studies from REF2014 to develop clear guidelines for the use of quantitative indicators in future impact case studies; in assessing the research environment, we recommend that there is scope for enhancing the use of quantitative data but that these data need to be provided with sufficient context to enable their interpretation; (18) the UK research community needs a mechanism to carry forward the agenda set out in this report; (19) the establishment of a Forum for Responsible Metrics, which would bring together research funders, HEIs and their representative bodies, publishers, data providers, and others to work on issues of data standards, interoperability, openness, and transparency; research funders need to increase investment in the science of science policy; and (20) one positive aspect of this review has been the debate it has generated. As a legacy initiative, the steering group is setting up a blog (www.ResponsibleMetrics.org) as a forum for ongoing discussion of the issues raised by this report.	The Metric Tide, although independent, was commissioned by the Minister for Universities and Science in 2014, in part to inform the REF used by universities across the country. Although the recommendations are sound, some of them might be perceived as being UK- centric. To what degree the recommendations might apply and be implemented on a global scale is unclear.
NAS (2015) [22]	United States	The US NAS and the Annenberg Retreat at Sunnylands convened this group of senior scientists ‘to examine ways to remove some of the current disincentives to high standards of integrity in science’. Incentives and rewards in academic promotion were included in this examination.	The authors indicate that if the current system does not evolve, there will be serious threats to the credibility of science. They state, ‘If science is to enhance its capacities to improve our understanding of ourselves and our world, protect the hard-earned trust and esteem in which society holds it, and preserve its role as a driver of our economy, scientists must safeguard its rigor and reliability in the face of challenges posed by a research ecosystem that is evolving in dramatic and sometimes unsettling ways’.	The authors ‘believe that incentives should be changed so that scholars are rewarded for publishing well rather than often. In tenure cases at universities, as in grant submissions, the candidate should be evaluated on the importance of a select set of work, instead of using the number of publications or impact rating of a journal as a surrogate for quality’. Incentives should also change to reward better correction (or self-correction) of science. It includes improving the peer-review process and reducing the stigma associated with retractions.	Because new incentives could be potentially damaging, authors ‘urge that each be scrutinized and evaluated before being broadly implemented’.
Nuffield Council on Bioethics (2014) [23]	UK	The Nuffield’s Culture of Scientific Research in the UK report ‘aimed to inform and advance debate about the ethical consequences of the culture of scientific research in terms of encouraging good research practice and the production of high quality science’. Through a combination of surveys and researcher engagement, feedback included ‘the perception that publishing in high impact-factor journals is the most important element in assessments for funding, jobs, and promotions is creating a strong pressure on scientists to publish in these journals’.	The feedback received cautioned maintaining the focus on JIFs—'This is believed to be resulting in important research not being published, disincentives for multidisciplinary research, authorship issues, and a lack of recognition for non-article research outputs’.	The report suggested actions for different stakeholders (funders, publishers and editors, research institutions, researchers, and learned society and professional bodies) to consider, principally, (1) improving transparency; (2) improving the peer-review process (e.g., by training); (3) cultivating an environment based on the ethics of research; (4) assessing broadly the track records of researchers and fellow researchers; (5) involving researchers in policy making in a dialogue with other stakeholders; and (6) promoting standards for high-quality science.	While the authors suggested different actions for various stakeholders, they emphasised that ‘a collective and coordinated approach is likely to be the most effective’. Such collaborative actions may be difficult to operationalise and implement.
REWARD (2014) [11]	Multinational	The Lancet commissioned a series, ‘Increasing value: Reducing Waste’, and follow-up conference to address the credibility of scientific research. The commissioning editors asked whether ‘the fault lie with myopic university administrations led astray by perverse incentives or with journals that put profit and publicity above quality?’	If the current bibliometric system is maintained, there is a real risk that scientists will be ‘judged on the basis of the impact factors of the journals in which their work is published’. Impact factors are weakly correlated with quality.	The REWARD series makes 17 recommendations covering a broad spectrum of stakeholders: (1) more research on research should be done to identify factors associated with successful replication of basic research and translation to application in healthcare and how to achieve the most productive ratio of basic to applied research; (2) research funders should make information available about how they decide what research to support and fund investigations of the effects of initiatives to engage potential users of research in research prioritisation; (3) research funders and regulators should demand that proposals for additional primary research are justified by systematic reviews showing what is already known, and increase funding for the required syntheses of existing evidence; (4) research funders and research regulators should strengthen and develop sources of information about research that is in progress, ensure that they are used by researchers, insist on publication of protocols at study inception, and encourage collaboration to reduce waste; (5) make publicly available the full protocols, analysis plans or sequence of analytical choices, and raw data for all designed and undertaken biomedical research; (6) maximise the effect-to-bias ratio in research through defensible design and conduct standards, a well-trained methodological research workforce, continuing professional development, and involvement of nonconflicted stakeholders; (7) reward with funding and academic or other recognition reproducibility practices and reproducible research, and enable an efficient culture for replication of research; (8) people regulating research should use their influence to reduce other causes of waste and inefficiency in research; (9) regulators and policy makers should work with researchers, patients, and health professionals to streamline and harmonise the laws, regulations, guidelines, and processes that govern whether and how research can be done, and ensure that they are proportionate to the plausible risks associated with the research; (10) researchers and research managers should increase the efficiency of recruitment, retention, data monitoring, and data sharing in research through the use of research designs known to reduce inefficiencies, and do additional research to learn how efficiency can be increased; (11) everyone, particularly individuals responsible for healthcare systems, can help to improve the efficiency of clinical research by promoting integration of research in everyday clinical practice; (12) institutions and funders should adopt performance metrics that recognise full dissemination of research and reuse of original datasets by external researchers; (13) investigators, funders, sponsors, regulators, research ethics committees, and journals should systematically develop and adopt standards for the content of study protocols and full study reports, and for data-sharing practices; (14) funders, sponsors, regulators, research ethics committees, journals, and legislators should endorse and enforce study registration policies, wide availability of full study information, and sharing of participant-level data for all health research; (15) funders and research institutions must shift research regulations and rewards to align with better and more complete reporting; (16) research funders should take responsibility for reporting infrastructure that supports good reporting and archiving; and (17) funders, institutions, and publishers should improve the capability and capacity of authors and reviewers in high-quality and complete reporting. There is a recognition of problems with academic reward systems that appear to focus on quantity more than quality. Part of the series includes a discussion about evaluating scientists on a set of best practices, including reproducibility of research findings, the quality of the reporting, complete dissemination of the research, and the rigor of the methods used.	There is little in the series about the relationship between the trustworthiness of biomedical research and hiring, promotion, and tenure of scientists. Similarly, the series does not propose an action plan for examining hiring, promotion, and tenure practices.
REF [24]	UK	There is a need to go beyond traditional quantitative metrics to gain a more in-depth assessment of the value of academic institutions.	Not being able to identify the societal value (e.g., public funding of higher education institutions and the impact of the research conducted) of academic institutions.	The REF is a new national initiative to assess the quality of research in higher education institutions assessing institutional outputs, impact, and environment covering 36 fields of study (e.g., law, economics, and econometrics). Outputs account for 65% of the assessment (i.e., ‘are the product of any form of research, published, such as journal articles, monographs and chapters in books, as well as outputs disseminated in other ways such as designs, performances and exhibitions’). Impact accounts for 20% of the assessment (e.g., ‘is any effect on, change or benefit to the economy, society, culture, public policy or services, health, the environment or quality of life, beyond academia’), and the environment accounts for 15% of the assessment (e.g., ‘the strategy, resources and infrastructure that support research’).	In the absence of a set of quantifiers for assessing the impact of the environment, it remains unclear if, based on the descriptions proposed, different evaluators would reach the same conclusions. This might be the case more for some criteria and less for others. The REF might stifle innovation and decrease collegiality across universities. Other limitations have been noted [44].
**Smaller group or individual proposals**					
Benedictus (UMC Utrecht, 2016) [25]	the Netherlands	The authors’ view, inspired by their related initiative, Science in Transition, is that ‘bibliometrics are warping science—encouraging quantity over quality’.	Focusing on meaningless bibliometrics is keeping scientists ‘from doing what really mattered, such as strengthening contacts with patient organizations or trying to make promising treatments work in the real world’.	To move away from using bibliometrics to evaluate scientists, the authors propose five alternative behaviours to assess: (1) managerial responsibilities and academic obligations; (2) mentoring students, teaching, and additional new responsibilities; (3) if applicable, a description of clinical work; (4) participation in organising clinical trials and research into new treatments and diagnostics; and (5) entrepreneurship and community outreach.	This is an intervention at the institutional level to try to change incentives and rewards. A novel set of criteria used to evaluate research and researchers was designed and has been introduced in a large academic medical centre. It needs to be studied how this new model is taken up in practice and whether it induces the intended effects.
Edwards (2017) [26]	US	Two engineers are concerned about the use of quantitative metrics to assess the performance of researchers.	The authors argue that continued reliance on quantitative metrics may lead to substantive and systemic threats to scientific integrity.	To deal with incentives and hypercompetition, the authors have proposed (1) that more data are needed to better understand the significance and extent of the problem; (2) that funding should be provided to develop best practices for assessing scientists for promotion, tenure, and hiring; (3) better education about scientific misconduct for students; (4) incorporating qualitative components, such as service to community, into PhD training programs; and (5) the need for academic institutions to reduce their reliance on quantitative metrics to assess scientists.	Some of the proposals like convening a panel of experts to develop guidelines for the evaluation of candidates or reframing the PhD as an exercise in ‘character building’ might be ineffective, confuse the panorama further, and not have support from a considerable part of the scientific community. Panels of experts are a notoriously unreliable and subjective source of evidence, they are exposed to groupthink, potential conflicts of interest, and reinforcing already existing biases. There is no reason to assume expertise is also associated with coming up with good practices. Given the financial hardships, gruesome work, and tough completion, the PhD program already is a Victorian exercise in ‘character building’.
Ioannidis (2014) [27]	US	This essay focuses on developing ‘effective interventions to improve the credibility and efficiency of scientific investigation’.	Currently, scientific publications are often ‘false or grossly exaggerated, and translation of knowledge into useful applications is often slow and potentially inefficient’. Unless we develop better scientific and publication practices, much of the scientific output will remain grossly wasted.	The author proposes 12 best practices to achieve truth and credibility in science. These include (1) large-scale collaborative research; (2) adoption of a replication culture; (3) registration; (4) sharing; (5) reproducibility practices; (6) better statistical methods; (7) standardisation of definitions and analyses; (8) more appropriate (usually more stringent) statistical thresholds; (9) improvement in study design standards; (10) stronger thresholds for claims of discovery; (11) improvements in peer review, reporting, and dissemination of research; and (12) better training of the scientific workforce. These best practices could be used as research currencies for promotion and tenure. The author provides examples of how these best practices can be used in different ways as part of the reward system for evaluating scientists.	The author acknowledges that ‘interventions to change the current system should not be accepted without proper scrutiny, even when they are reasonable and well intended. Ideally, they should be evaluated experimentally’. Many of the research practices lack sufficient empirical evidence as to their worth.
Mazumdar (2015) [28]	US	This group was focused on ways to assess team science—often the role biostatisticians find themselves in. Their view is that ‘those responsible for judging academic productivity, including department chairs, institutional promotion committees, provosts, and deans, must learn how to evaluate performance in this increasingly complex framework’.	Concentrating on traditional metrics ‘can substantially devalue the contributions of a team scientist’.	To assess the research component of a biostatistician as part of a team collaboration, the authors propose a flexible quantitative and qualitative framework involving four evaluation themes that can be applied broadly to appointment, promotion, and tenure decisions. These criteria are: design activities, implementation activities, analysis activities, and manuscript reporting activities.	The authors state, ‘The paradigm is generalizable to other team scientists’. However, ‘because team scientists come from many disciplines, including the clinical, basic, and data sciences, the same criteria cannot be applicable to all’. One limitation is the potential gaming of such a flexible scheme.
Ioannidis PQRST (2014) [29]	US	The authors of this paper state the problem as ‘scientists are typically rewarded for publishing articles, obtaining grants, and claiming novel, significant results’.	The authors note, ‘However, emphasis on publication can lead to least publishable units, authorship inflation, and potentially irreproducible results’. In short, this type of assessment might tarnish science and how scientists are evaluated.	To reduce our reliance on traditional quantitative metrics for assessing and rewarding research, the authors propose a best practice index—PQRST—revolving around productivity, quality, reproducibility, sharing, and translation of research. The authors also propose examples on how each item could be operationalised, e.g., for productivity; examples include number of publications in the top tier percentage of citations for the scientific field and year, proportion of funded proposals that have resulted in ≥1 published reports of the main results, and proportion of registered protocols that have been published 2 years after the completion of the studies. Similarly, one could count the proportion of publications that fulfill ≥1 quality standards; proportion of publications that are reproducible; proportion of publications that share their data, materials, and/or protocols (whichever items are relevant); and proportion of publications that have resulted in successful accomplishment of a distal translational milestone, e.g., getting promising results in human trials for interventions tested in animals or cell cultures, or licensing of intervention for clinical trials.	The authors acknowledge that some indicators require building new tools to capture them reliably and systematically. For quality, one needs to select standards that may be different per field/design and this requires some consensus within the field. There is no wide-coverage automated database currently for assessing reproducibility, sharing, and translation, but proposals are made on how this could be done systematically and who might curate such efforts. Focusing on the top, most influential publications may also help streamline the process.
Nosek (2015) [30]	US	The authors state the problem as truth versus publishability—'The real problem is that the incentives for publishable results can be at odds with the incentives for accurate results. This produces a conflict of interest. The conflict may increase the likelihood of design, analysis, and reporting decisions that inflate the proportion of false results in the published literature’.	With the perverse ‘publish or perish’ mantra, the authors argue that authors may feel compelled to fabricate their results and undermine the integrity of science and scientists. ‘With flexible analysis options, we are more likely to find the one that produces a more publishable pattern of results to be more reasonable and defensible than others’.	The authors propose a series of best practices that might resolve the aforementioned conflicts. These best practices include restructuring the current incentive/reward scheme for academic promotion and tenure, use of reporting guidelines, promoting better peer review, and journals devoted to publishing replications or statistically negative results.	The analyses and proposed actions and interventions are very clear and generate awareness. To have even more effect, these actions should be taken up by leaders in academia and institutions. Actions by funding agencies may also be required to set proper criteria for their reviewers of grant proposals.
**Journal proposals**					
*eLife* (2013) [31]	US	The editors of *eLife* are concerned about the ‘widespread perception that research assessment is dominated by a single metric, the journal impact factor (JIF)’. They are interested in reforming these evaluations using different metrics.	The editors state, ‘The focus on publication in a high impact-factor journal as the prize also distracts attention from other important responsibilities of researchers—such as teaching, mentoring and a host of other activities (including the review of manuscripts for journals!). For the sake of science, the emphasis needs to change’.	To help counter these problems, the editors discuss several options, such as repositories for sharing information: Dryad for datasets; Figshare for primary research, figures, and datasets; and Slideshare for presentations. Altmetric.com, Impact Story, and Plum Analytics can be used to aggregate media coverage, citation numbers, and social web metrics. Taken together, such information is likely to provide a broader assessment of the impact of research well beyond that of the JIF.	While exemplary, it is unclear how widespread these initiatives will become and whether there are implementation hurdles. To have broader impact, similar initiatives need to be endorsed and implemented in thousands of journals.
*Nature* (2016) [32]	UK	The journal’s perspective is that ‘metrics are intrinsically reductive and, as such, can be dangerous. Relying on them as a yardstick of performance, rather than as a pointer to underlying achievements and challenges, usually leads to pathological behaviour. The journal impact factor is just such a metric’.	Relying on the JIF will maintain the aforementioned problems.	To help combat these problems, the journal has proposed two solutions: first, ‘applicants for any job, promotion or funding should be asked to include a short summary of what they consider their achievements to be, rather than just to list their publications’. Second, ‘journals need to be more diverse in how they display their performance’.	While the use of diverse metrics is a positive step, they are not helpful for researchers across different disciplines. For example, Altmetrics does not have field-specific scores yet. It is difficult to know what these alternative metrics mean and how they should be considered within a researcher’s evaluation portfolio.
**Quantitative proposals**					
RCR (2015) [33]	US	These authors were interested in developing a scientifically rigorous alternative to the current perverse prestige of the JIF for assessing the merit of publications and, by association, scientists.	The authors list a number of problems with maintaining current bibliometrics. Many of these are echoed in other reports/papers in this table.	The authors report on the development and validation of the RCR metric. The RCR ‘is based upon the novel idea of using the co-citation network of each article to field- and time-normalize by calculating the expected citation rate from the aggregate citation behavior of a topically linked cohort’. The article citation rate is the numerator and the average citation rate is the denominator.	More independent research is needed to examine the relation between the RCR and other metrics and the predictive validity of the RCR as well as whether it probes untoward consequences, such as gaming or endowing questionable research practices. A recent paper disputes the validity of the RCR by raising several concerns regarding the calculation algorithm [51].
Journal citation distributions (2016) [34]	International	The JIF is a poor summary of raw distribution of citation numbers from a given journal, because that distribution is highly skewed to high values.	The JIF says little about the likely citation numbers of any single paper in a journal, let alone other dimensions of quality that are poorly captured by citation counts.	The authors proposed using full journal citation distributions or nonparametric summaries (e.g., IQR) and reading of individual papers to evaluate both the papers and journals.	It is not clear how to use full JCRs to evaluate either a journal or a specific paper or what summaries of the JCR are most informative. Citation counts do not capture the reason for the citation.
R-index (2015) [35]	Canada/Denmark	Peer review is under many threats. For one, we are fast approaching a situation in which the number of manuscripts requiring peer review will outstrip the number of available peer reviewers. Peer reviewing, while an essential part of the scientific process, remains largely undervalued when assessing scientists.	The authors have proposed a quantitative metric to gauge the contributions towards peer reviewing by researchers. ‘By giving citeable academic recognition for reviewing, R-index will encourage more participation with better reviews, regardless of the career stage’.	The R-index is calculated as ‘Each journal, *j*, will disclose their annual list of reviewers, *i*, and the number of papers they reviewed, *nj*. For each kth paper in a given journal *j*, the total number of words, wkj, is multiplied by the square root of the journal’s impact factor, IFj. This product is weighted by the editor’s feedback on individual revisions, which is given by a score of excellence, skj, ranging from 0 (poor quality) to 1 (exceptionally good quality)’. Traditionally, peer review is given little weight in assessing scientists. This metric might provide an opportunity to more realistically credit the contributions of peer reviewing to the larger scientific community. This metric could be an alternative to the usual sole focus on JIF.	The index is exceedingly complex; interpretation is not intuitive and requires standardised inputs that are not currently gathered at journals. It has not yet been applied to multiple journals and relies partly on IF.
S-index (2017) [36]	US	The current system, by not according any credit to the production of data that others then use in publications, disincentivises data sharing and the ability to assess research reproducibility or make new scientific contributions from the shared data.	The authors have proposed a metric to offer authors’ a measurable incentive to share their data with other researchers.	The S-index is in essence an H-index for publications that uses scientists’ shared data but does not include those researchers as authors. It counts the number of such publications, *N*, that get more than *N* citations. Promotion and tenure committees could use this metric as a complement to direct citation counts.	This has not been applied in practice. It is not clear how to assign sharing credit to initiatives with many authors. There is no established method to track use of datasets; it relies on citation counts.

**Abbreviations:** ACUMEN, Academic Careers Understood through Measurement and Norms; DOI, digital object identifier; DORA, Declaration on Research Assessment; GEP, Good Evaluation Practices; *i*, annual list of reviewers; ID, identifier; IF, impact factor; IFj, journal’s impact factor; IQR, interquartile range; ISNI, International Standard Name Identifier; *j*, journal; JIF, journal impact factor; *N*, number of publications that use a scientist’s shared data but does not include those researchers as authors; NAS, National Academy of Sciences; *nj*, number of papers reviewed; PQRST, Productive, high-Quality, Reproducible, Shareable, and Translatable; RCR, relative citation ratio; REF, Research Excellence Framework; REWARD, Reduce research Waste And Reward Diligence; UMC, Utrecht Medical Centre; wkj, total number of words.

We invited 23 academic leaders: deans of medicine (e.g., Oxford), public and foundation funders (e.g., National Institutes of Health [NIH]), health policy organisations (e.g., Science Policy, European Commission; Belgium), and individual scientists from several countries. They were selected based on contributions to the literature on the topic and representation of important interests and constituencies. Twenty-two were able to participate (see S1 Table for a complete list of participants and their affiliations). Prior to the meeting, all participants were sent the results of a selected review of the literature distilled into a table (see Table 1) and several selected readings.

Table 1 served as the basis for an initial discussion about the problems of assessing scientists. This was followed by discussions of possible solutions to the problems, new approaches for promotion and tenure committees, and implementation strategies. All discussions were recorded, transcribed, and read by five coauthors. For this, six general principles were derived. This summary was then shared with all meeting participants for additional input.

## Results

We included a list of 21 documents [11,17–36] in Table 1. There has been a burgeoning interest in assessing scientists in the last 5 years (publication year range: 2012–January 2017). An almost equal number of documents originated from the US and Europe (one also jointly from Canada). We divided the documents into four categories: large group efforts (e.g., the Leiden Manifesto [20]), smaller group or individual efforts (e.g., Ioannidis and Khoury’s Productive, high-Quality, Reproducible, Shareable, and Translatable [PQRST] proposal [29]); journal activities (e.g., *Nature* [32]); and newer quantitative metric proposals (e.g., journal citation distributions [34]).

We interpreted all of the documents to describe the problems of assessing science and scientists in a similar manner. There is a misalignment between the current problems in research. The right questions are not being asked; the research is not appropriately planned and conducted; and when the research is completed, results remain unavailable, unpublished, or get selectively reported; reproducibility is lacking as is evidence about how scientists are incentivised and rewarded. We interpreted that several of the documents pointed to a disconnect between the production of research and the needs of society (i.e., productivity may lack translational impact and societal added value). We paraphrased the views expressed across several of the documents: ‘we should be able to improve research if we reward scientists specifically for adopting behaviours that are known to improve research’. Finally, many of the documents described the JIF as an inadequate measure for assessing the impact of scientists [19,20,29]. The JIF is commonly used by academic institutions [4,5,37] to assess scientists, although there are efforts to encourage them not to do so. Not modifying the current assessment system will likely result in the continued bandwagon behaviour that has not always resulted in positive societal behaviour [25,38]. Acting now to consider modifying the current assessment system might be seen as a progressive move by current scientific leaders to improve how subsequent generations of scientists are evaluated.

### Large group proposals for assessing scientists

Nine large group efforts were included [11,17–24] (see Table 1), representing different stakeholder groups. The Leiden Manifesto [20] and the Declaration on Research Assessment (DORA) [19] were both developed at academic society meetings and are international in their focus, whereas the Metric Tide [21] was commissioned by the UK government (operating independently) and is more focused on the UK academic marketplace.

The Leiden Manifesto authors felt that research evaluation should positively support the development of science and its interactions with society. It proposes 10 best practices, for example, that expert qualitative assessment should take precedence, supported by the quantitative evaluation of a researcher using multiple indices. Several universities have recently pledged to adopt these practices [39].

The San Francisco DORA, which is also gaining momentum [40–42], was first developed by editors and publishers and focuses almost exclusively on the misuse of the JIF. DORA describes 17 specific practices to diminish JIF dependence by four stakeholder groups: scientists, funders, research institutions, and publishers. DORA recommends focusing on the content of researchers’ output, citation of the primary literature, and using a variety of metrics to show the impact. Within evidence-based medicine, systematic reviews are considered stronger evidence than individual studies. DORA’s clarification about citations and systematic reviews might facilitate its endorsement and implementation within faculties of medicine.

The National Academy of Sciences proposed that scientists should be assessed for the impact of their work rather than the quantity of it [22]. Research impact is also raised as an important new assessment criterion in the UK’s Research Excellence Framework (REF) [24], an assessment protocol in which UK higher education institutions and their faculty are asked to rate themselves on three domains (outputs, impact, and environment) across 36 disciplines. These assessments are linked to approximately a billion Great Britain Pounds (GBPs) of annual funding to universities, of which 20% (to be increased to 25% in the next round) is based on the impact of the faculty member’s research. The inclusion of assessing impact (e.g., through case studies) has fostered considerable discussion across the UK research community [43]. Some argue it is too expensive, that pitting universities against each other can diminish cooperation and collegiality, that it does not promote innovation, and that it is redundant [44]. The Metric Tide, which has been influential in the UK as it relates to that country’s REF, made 20 recommendations related to how scientists should be assessed [21]. It recommends that universities should be transparent about how faculty assessment is performed and the role that bibliometrics plays in such assessment, a common theme across many of the efforts (e.g., [19,20]).

### Individual or small group proposals for assessing scientists

Six smaller group or individual proposals were included [25–30] (see Table 1). For example, Mazumdar and colleagues discussed the importance of rewarding biostatisticians for their contributions to team science [28]. They proposed a framework that separately assesses biostatisticians for their unique scientific contributions, such as the design of a grant application and/or research protocol, and teaching and service contributions, including mentoring in the grant application process.

Ioannidis and Khoury have proposed scientist assessments revolving around ‘PQRST’: Productivity, Quality, Reproducibility, Sharing, and Translation of research [29]. Benedictus and Miedema describe approaches currently being used at the Utrecht Medical Centre, the Netherlands [25]. For the Utrecht assessment system, a set of indicators was defined and introduced to evaluate research programs and teams that mix the classical past-performance bibliometric measures with process indicators. These indicators include evaluation of leadership, culture, teamwork, good behaviours and citizenship, and interactions with societal stakeholders in defining research questions, the experimental approaches, and the evaluation of the ongoing research and its results. The latter includes semi-hybrid review procedures, including peers and stakeholders from outside academia. The new assessment for individual scientists is complemented by a semi-qualitative assessment in a similar vein for our multidisciplinary research programs. Taking a science policy perspective, these institutional policies are currently being evaluated with the Centre for Science and Technology Studies (CSTS) in Leiden by investigating how evaluation practices reshape the practice of academic knowledge production.

Not surprisingly, many of these proposals and individual efforts overlap. For example, the Leiden Group’s fourth recommendation, ‘Keep data collection and analytical processes open, transparent and simple’, is similar to DORA’s 11th specific (of 17) recommendation, ‘Be open and transparent by providing data and methods used to calculate all metrics’. Some groups use assessment tools as part of their solutions. The Academic Careers Understood through Measurement and Norms (ACUMEN) group produced a weblike document system [17], whereas the Mazumdar group created checklists [28].

Groups targeted different groups of stakeholders. The Nuffield Council on Bioethics targeted funders, research institutions, publishers and editors, scientists, and learned societies and professional bodies [23], as did the Reduce research Waste And Reward Diligence (REWARD) Alliance, who added regulators as one of their target groups [11].

Most of the proposals were aspirational in that they were silent on details of how exactly faculty assessment committees could implement their proposals, what barriers to implementation existed, and how to overcome them. Integrating behavioural scientists and implementation scientists into implementation discussions would be helpful. Ironically, most of the proposals do not discuss whether or how to evaluate the impact of adopting them.

### Journal proposals for assessing scientists

Journals may not appear to be the most obvious group to weigh in on reducing the reliance on bibliometrics to assess scientists. Traditionally, they have been focused on (even obsessed with) promoting their JIFs. Yet, some journals are beginning to acknowledge the JIF’s possible limitations. For example, the *PLOS* journals do not promote their JIFs [45]. BioMed Central and Springer Open have signed DORA, stating, ‘In signing DORA we support improvements in the ways in which the output of scientific research is evaluated and have pledged to “greatly reduce emphasis on the journal Impact Factor as a promotional tool by presenting the metric in the context of a variety of journal-based metrics”’ [46].

We included two journal proposals [31,32]. *Nature* has proposed, and in some cases implemented, a broadening of how they use bibliometrics to promote their journals [32]. We believe a reasonable short-term objective is for journals to provide more potentially credible ways for scientists to use journal information in their assessment portfolio. *eLife* has proposed a menu of alternative metrics that go beyond the JIF, such as social and print media impact metrics, that can be used to complement article and scientist assessments [31].

Journals can also be an instrument for promoting best publication practices among scientists, such as data sharing, and academic institutions can focus on rewarding scientists for employing those practices rather than quantity of publications alone. Reporting biases, including publication bias and selective outcome reporting, are prevalent [14]. A few journals, particularly in the psychological sciences, have started using a digital badge system that promotes data sharing, although there has been some criticism of them [47]. The journal *Psychological Science* has evaluated whether digital badges result in more data sharing [48]. Such badges may potentially be used for assessing scientists based on whether they have adhered to these good publication practices. As of mid-2017, 52 mostly psychology journals have agreed to review and accept a paper at the protocol stage if a ‘registered report’ has been recorded in a dedicated registry [49]. If such initiatives are successful, assessors could reward scientists for registering their protocols, as with clinical trials and systematic reviews they can currently monitor timely registration in one of several dedicated registries.

### Proposals to improve quantitative metrics

There are many efforts to improve quantitative metrics in the vast and rapidly expanding field of scientometrics. We included four proposals [33–36]. An influential group including editors (*Nature*, *eLife*, *Science*, *EMBO*, *PLOS*, *The Royal Society*), a scientometrician, and an advocate for open science proposed that journals use the journal citation distribution instead of the JIF [36]. This allows readers to examine the skewness and variability in citations of published articles. Similarly, the relative citation ratio (RCR) or the Source Normalized Impact per Paper (SNIP) have been proposed to adjust the citation metric by content field, addressing one of the JIF’s deficiencies [19]. Several proposals have recognised the need for field-specific criteria [20,21]. However, the value of different normalisations needs further study [50] and there has been criticism of the proposed RCR [51]. There are currently dozens of citation indicators that can be used alone or in combination [52]. Each has its strengths and weaknesses, including the possibility for ‘gaming’ (i.e., manipulation by the investigator).

Besides citation metrics, there is also increasing interest in developing standardised indicators for other features of impact or research practices, for example, alternative metrics, as discussed above. For example, Twitter, Facebook, or lay press discussions might indicate influence on or accessibility to patients. However, alternative metrics can also be gamed and social media popularity may not correlate with scientific, clinical, and/or societal benefit. An ‘S-index’ has been proposed to assess data sharing [36], albeit with limitations (see Table 1).

### Principles for assessing scientists

Six general principles emerged from the discussions, each with research and policy implications (see Table 2). Several have been proposed previously [53].

**Table 2:** Key principles, participant dialogue, and research and policy implications when assessing scientists.

Number	Key principles	Participant dialogue	Research implications	Policy implications
1	Addressing societal needs is an important goal of scholarship.	There was a discussion on the need for research and faculty to look outwardly and not solely focus inwardly. The inward obsession, in which researchers have to struggle hard to secure and advance their own career, has resulted in bandwagon behaviour that has not always had positive societal impact. Participants also discussed the need to expand who can meaningfully contribute to scientific ideas. The public and patients need to be included as part of this process.	There is a need to develop mechanisms to evaluate whether research helps society. This is particularly important for scientists working on applied science rather than blue-sky discovery, the immediate and midterm societal impact of which may be more difficult to discern. Innovative assessment practices in applied settings might reward the professional motivations of young researchers when their research efforts more closely align with solving the societal burden of disease(s).	Academic institutions are part of the broader society and their faculty should be evaluated on their contributions to the local community and more broadly. For example, universities could ask their faculty assessment committees to focus their assessments on whether patients were involved in selecting outcomes used in clinical trials or whether faculty shared their data, code, and materials with colleagues. Most bibliometric indicators do not necessarily gauge such contributions, and faculty assessment criteria need to be broadened to reward behaviours that impact society.
2	Assessing faculty should be based on responsible indicators that reflect fully the contribution to the scientific enterprise.	Besides bibliometric indices (which need to be used in their optimal form, minimising their potential for gaming), several responsible indicators for assessing faculty (RIAS’s) were discussed (see main text). A dashboard was considered an appealing option to implement RIAS’s across academic institutions. Supplemental indicators could be added for institutions interested in evaluating additional specific values and/or behaviours. Participants discussed the merits of linking any RIAS dashboard to university ranking schemes (e.g., Times Higher Education World University Rankings). Participants felt strongly that some of the currently used metrics are imperfect and provide an inaccurate assessment of the value of scientists’ contributions to science. They may also promote poor behaviour—particularly the JIF, which needs to be de-emphasised.	Identifying and collating current promotion and tenure criteria across academic institutions is essential to provide baseline knowledge, against which initiatives and/or policy changes can be evaluated. Assuming OA publishing is deemed an important RIAS by a university when evaluating their scientists, then the university could gauge the fraction of OA publications prior to and following implementing this RIAS. A comparison of whether the OA papers have a wider reach to the local community, and more broadly (more citations, Altmetrics), could also be evaluated. Prior to such an implementation, the university would need to expand its OA fund for faculty use. This could be achieved through use of endowment funds. Some responsible indicators are new and data are needed as to whether they are robust and informative ways to assess faculty. Academic institutions and funders need to support such efforts. Additional measures of scientific value when assessing faculty should be elicited from faculty. Academic institutions may still use bibliometrics indices to assess their faculty, with appropriate understanding of their strengths and limitations. Evidence from scientometrics on bibliometric indicators is far stronger than for any of the new RIAS, and such evidence should be used to pick the most appropriate bibliometric indicators. For all indicators, old and new, it is essential to study whether they are associated eventually with better science.	Leadership is critical to moving this initiative forward. This could start by university leaders asking their respective assessment committees to review and make available current criteria used to assess scientists. The results could be shared with faculty members, as could their opinions about the relevance of the current criteria and suggestions for potential new and evidence-based assessment criteria. Such discussions could also be held with the local community to address whether the assessments reflect their values. Funders should implement a policy to explicitly indicate that their grant assessment committees will de-emphasise using poor indicators (e.g., JIF) and favour better ones, including validated new RIAS’s. Funders and/or academic institutions could pay for monitoring and making it openly available through independent audit and feedback. Like funders, promotion and tenure committees at academic institutions should implement a similar policy when assessing their faculty. Institutions can signal such a commitment by signing one of several initiatives (e.g., the Leiden Manifesto). The signal and any subsequent implementation could be monitored through audit and feedback. University leaders could launch a prize funded through their endowments for the best promotion and tenure committee implementation strategies, judged by an independent panel. All of these initiatives might have a greater chance of success if at the outset university leaders explicitly indicated that if the policies are not implemented and adhered to (gauged through audit and feedback), they would agree to a reprimand of some kind.
3	Publishing all research completely and transparently, regardless of the results, should be rewarded.	Participants discussed the need to reduce the problems associated with reporting biases, including publication bias. Making all research available, either through formal publication or other initiatives, such as preprints and OA repositories, is very valuable and faculty should be rewarded for such behaviour. The value of reporting guidelines to improve the completeness and transparency of reporting research was also discussed.	Some journals are developing innovative ways, such as registered reports, to help ensure that all results are reported. Audit and feedback might augment the uptake of any new initiative to promote better reporting of research and subsequent publication. Any new initiative needs to include an evaluation component, ideally using experimental methods, to ascertain whether it achieves its intended effect.	Funders and academic institutions are well positioned to implement policies to promote more complete reporting of all research. Rewarding faculty for registering their planned studies and publishing/depositing their completed studies is essential. Academic institutions should consider linking ethics approval with study registration. Promotion and tenure assessment should reward all efforts to make research available and transparently reported. Journals should endorse and implement reporting guidelines that facilitate complete and transparent reporting.
4	The culture of Open Research needs to be rewarded.	Participants agreed on the need to value more open research (i.e., including sharing of data, protocols, software, code, materials, and other research tools). Open research is essential to facilitate reproducibility. Behaviours that promote open research should be rewarded.	Open research is becoming a more widely accepted cultural norm. Initiatives such as TOP [59] guidelines are helping journals promote open science practices. All initiatives need to be evaluated as to whether they are achieving their intended goal.	Funders, journals, and academic institutions should promote open research behaviours and reward them appropriately. This is likely a shared principle across stakeholders. A strong signal concerning its importance could be based on joint implementation across funders, journals, and academic institutions. For example, audit and feedback (e.g., Trialtracker tool [61]) of registration of study protocols, the use of journals with registered reports [49], and reporting completed clinical trials benefit all stakeholders. Journals could commit to and fund independent audit and feedback of TOP, the results of which are made publicly available. Perhaps a TOP score could become an important criterion when considering submission to a journal. Like the other principles, innovative leadership is essential for implementation. Similarly, for such policies to be effective, there need to be explicit rewards for reaching milestones and consequences in cases in which implementation has not occurred.
5	It is important to fund research that can provide an evidence base to inform optimal ways to assess science and faculty.	Newer fields of investigation, such as journalology (publication science) and meta-research, help generate data on optimal ways to assess faculty. There was a strong call to promote experimental paradigms to evaluate initiatives.	There have been several initiatives to reform how faculty is assessed for promotion and tenure. Any new initiative needs to have an evaluation component built into the process—does the new initiative (i.e., intervention) have its intended effect? There are some initial data suggestive of an association between data sharing and increased citations [77]. A more robust evaluation might include a cluster-randomised trial whereby different universities would be allocated to a data-sharing policy or standard university practice. One outcome could be a comparison of the subsequent number of citations between universities. Such data are vital to understanding how best to design, assess, and implement any such initiatives in the future.	Funding agencies should set aside funding to promote evaluations for assessing faculty and research on research (see text for examples). In the same way that funders have priority and/or specific areas of interest (e.g., cancer), they should also be interested in how well they (and their grantees) meet best publication practices for the money they are spending. Specific research calls should be made on an ongoing basis. Such a policy would signal the importance funders place on how scientists are evaluated.
6	Funding out-of-the-box ideas needs to be valued in promotion and tenure decisions.	Participants held it was important to promote grant applications without specific aims, securing support for pursuing a broader investigative agenda.	Different models on how to support out-of-the-box ideas need to be evaluated comparatively, using appropriate midterm and long-term outcome indicators.	Funders need to promote opportunities for blue-sky thinking without any immediate return on investment and promote success stories. This might be achieved through stable longer-term, perhaps in the 4- to 8-year range, funding of scientists at different career stages. Such funding could be a specific fraction of the funder’s total budget. The Canadian Institutes of Health Research’s Foundation scheme and the Australia’s National Health and Medical Research Council Early Career Fellowships are examples of this type of funding. The Human Genome Project might provide a useful model to fund these programs. It invested 1% of its budget in the Ethical, Legal and Social Implications Research Program, with enormous payoff.

**Abbreviations:** JIF, journal impact factor; OA, open access; RIAS, responsible indicator for assessing scientists; TOP, transparency and openness promotion.

The first principle is that contributing to societal needs is an important goal of scholarship. Focusing on research that addresses the societal need and impact of research requires a broader, outward view of scientific investigation. The principle is based on academic institutions in society, how they view scholarship in the 21st century, the relevance of patients and the public, and social action [10]. If promotion and tenure committees do not reward these behaviours, or penalise practices that diminish the social benefit of research, maximal fulfillment of this goal is unlikely [25].

The second principle is that assessing scientists should be based on evidence and indicators that can incentivise best publication practices. Several new ‘responsible indicators for assessing scientists’ (RIAS’s) were proposed and discussed. These include assessing registration (including registered reports); sharing results of research; reproducible research reporting; contributions to peer review; alternative metrics (e.g., uptake of research by social media and print media) assessed by several providers, such as Altmetric.com; and sharing of datasets and software assessed through Impact Story [54]). Such indicators should be measured objectively and accurately, as publication and citation tools do currently. Some assessment items, such as reference letters from colleagues and stakeholders affected by the research, cannot be converted into objective measurements, but one may still formally investigate their value [55].

As with any new measures, RIAS characteristics need to be studied in terms of ease of collection, their frequencies and distributions in different fields and institutions, the kind of systems needed to implement them, and their usefulness in both evaluation and modifying researcher behaviours and the extent to which each may be gamed. Different institutions could and should experiment with different sets of RIAS’s to assess their feasibility and utility. Ultimately, if there were enough consensus around a core set, institutional research funding could be tied to their collection, such as underlies successful implementation of Athena Scientific Women's Academic Network (SWAN) for advancing gender equity, which has been highly successful in the UK [56].

One barrier to implementation of any RIAS scheme is whether it would affect current university rankings (e.g., Times Higher Education World University Rankings). Productivity, measured in terms of publication output, is an important input into such rankings. Participants felt that any RIAS dashboard could be included in or as an alternative to university ranking schemes. However, these ranking systems are themselves problematic; the Leiden CSTS has recently proposed 10 principles regarding the responsible use of such ranking systems [57].

The third principle is that all research should be published completely and transparently, regardless of the results. Academic institutions could implement policies in the promotion process to review complete reporting of all research, and/or penalise noncompleted or nonpublished research—particularly clinical trials, which must be registered. For nonclinical research, participants discussed the need to reward other types of openness, such as sharing of datasets, materials, software and methods used, and explicit acknowledgment of their exploratory nature, when appropriate [58]. Finally, finding fair ways to reward team endeavors is critical, given the growing collaborative nature of research, which bibliometrics cannot properly assess. For example, some promotion and tenure committees largely disregard work for which the faculty candidate is not the first or senior author [4]. Conversely, citation metrics that do not correct for multiple coauthorship and thus authors who are just appearing in long author mastheads can result in inappropriately high citation metrics.

The fourth principle relates to openness—facilitating dissemination and use of research data and results by others. Researchers can share their data, procedures, and code in various ways, such as in open access repositories. Some journals are supporting this process by endorsing and implementing the transparency and openness promotion (TOP) guidelines [59]. Groups that rank universities can also support this principle by sharing the underlying data used to make their assessments.

The fifth principle requires investing in research to provide the necessary evidence to guide the development of new assessment criteria and to evaluate the merits of existing ones. Funders are the ones to make such investments and some, particularly in Europe (e.g., Netherlands Organisation for Scientific Research), have already started.

The final principle involves rewarding researchers for intellectual risk-taking that might not be reflected in early successes or publications. The need for a young researcher to obtain their own funding early often results in a conservatism that is inimical to groundbreaking work at a time when they might be the most creative. Changing assessments to evaluate and reward such hypotheses might encourage truly creative research. It is also possible to conduct some forms of research with limited funding [60].

### Implementation

A challenge introducing any of these principles, or other new ideas, is how best to operationalise them. The TrialsTracker tool [61] enables institutions from around the world (with more than 30 trials) to monitor their trial reporting. Although the tool has limitations [62], it has a low barrier to implementation and provides a useful and easy starting point for audit and feedback. Promotion and tenure committees could receive such data as part of annual faculty assessment. They could also ask scientists to modify their CVs to incorporate information about registration through indicating the name and registration number of the registry, whether they have participated in a journal’s registered reports program, and a citation of the completed and published study. For each new initiative, it is important to generate evidence, ideally from experimental studies, on whether it leads to better outcomes.

Participants also discussed that efforts to reward good, rigorously conducted evaluation should not come at the expense of stifling creative ‘blue-sky’ research primarily aimed at understanding biologic processes.

### Moving forward

Current systems reward scientific innovation, but if we want to improve research reproducibility, we need to find ways to reward scientists who focus on it [63–65]. A scientist who detects analytical errors in published science and works with the authors to help correct the error needs to have such work recognised. This benefits the original scientists, participants in the original research, the journal publishing the original research, the field, and society. The authors of the original report could include documentation, perhaps in the form of an impact letter, attesting to the value of the reproducibility efforts, which could be included in the evaluation portfolio.

High-quality practice guidelines are evidence based, typically using systematic reviews as one of their foundational building blocks. We likely need to develop similar evidence-based approaches when assessing scientists. Despite some criticism, the UK’s REF is a step in this direction [66,67]. The metrics marketplace is large and confusing. Institutions can choose or pick metrics with an evidence base and endorsed by reputable organizations: the NIH sponsored development of the RCR and the SNIP was developed by Leiden University’s CSTS. Regardless of which approach is adopted, evidence on the accuracy, validity, and impact of indicators is necessary.

If best practices for appraising scientists can be identified, achieving widespread adoption will be a major challenge [68]. Ultimately, this may depend on institutional values, which might be elicited from the institution’s faculty. Junior faculty may put a high value on open access publications [69]. If open access were to become part of RIAS [70] and included in faculty assessments, the institution would need to support open access fees. Committed support from leadership and senior faculty would be needed to implement the policy points discussed in Table 2. Finally, implementation for some of the six principles should be easier if stakeholders worked collaboratively, so as not to work at cross-purposes.

Institutional promotion and tenure committee guidelines are not easily available outside researchers, although there is an effort underway to compile them [71]. If institutions made them available, this information could be used as a baseline to gauge changes in criteria and also to disseminate institutional innovations. We also call for institutions to examine their own awards and promotion practices to understand how their high-level criteria are being operationalised and to see the effect of criteria such as counting the number of first and last author publications. Funders can also make widely available what criteria they use from grant applications to assess scientists.

Whether implemented at the local or national level, changes in assessment criteria should be fully documented and made openly available. Institutions making changes to their promotion and tenure criteria and faculty assessment should implement an evaluation component as part of the process. Evaluations using experimental approaches are likely to provide the most internally valid results and may offer greater generalizability. Stepped wedge designs [72] or interrupted time series [73] might be appropriate for assessing the effects of individual or multiple department promotion and tenure committees’ uptake of new assessment criteria for scientists, together with audit and feedback. These data can inform the development of new systems [74].

A few funders have set aside specific funding streams to fund research on research. The Dutch Medical Research Council has established a funding stream called ‘Responsible Research Practices’, which recently awarded eight grants. A central aspect of the approach taken by Mark Taylor, the new Head of Impact for the UK National Institute for Health Research (NIHR) is to ‘… give patients more tools to help shape the research future, their future itself’ [75]. Widening the spectrum of funders making such investments would serve as a powerful message about their values to both researchers and institutions.

We did not complete a systematic review (including other fields, such as engineering), the results of which may provide additional knowledge to what we have reported here. The principles are not comprehensive. They reflect discussions between participants. This field of research and researcher assessments is currently fragmented with an uneven evidence base that has an enormous volume of publications on some topics (e.g., scientometrics) and little evidence on others. As the field grows, we hope it generates stronger data to help inform decision-making. The research implications in Table 2 can be a starting point for investing more heavily in providing that evidence. However, when a new assessment measure is developed and evaluated, it may fall prey to Goodhart’s law [76] (i.e., it ceases as a valid measurement when it becomes an optimisation target); the unintended effects of individuals or institutions trying to optimise these measures will require close attention.

How we evaluate scientists reflects what we value most—and don't—in the scientific enterprise and powerfully influences scientists’ behaviour. Widening the scope of activities worthy of academic recognition and reward will likely be a slow and iterative process. The principles here could serve as a road map for change. While the collective efforts of funders, journals, and regulators will be critical, individual institutions will ultimately have to be the crucibles of innovation, serving as models for others. Institutions that monitor what they do and the changes that result would be powerful influencers of the shape of our collective scientific future.

## Supporting information

S1 Table Name, portfolio, and affiliation of workshop organisers and participants. (DOCX)

## Abbreviations

ACUMEN: Academic Careers Understood through Measurement and Norms
CSTS: Centre for Science and Technology Studies
CV: curriculum vita
DOI: digital object identifier
DORA: Declaration on Research Assessment
GBP: Great Britain Pound
GEP: Good Evaluation Practices
ISNI: International Standard Name Identifier
JIF: journal impact factor
METRICS: Meta-Research Innovation Center at Stanford
NAS: National Academy of Sciences
NIH: National Institutes of Health
NIHR: National Institute for Health Research
OA: open access
PQRST: Productive, high-Quality, Reproducible, Shareable, and Translatable
RCR: relative citation ratio
REF: Research Excellence Framework
REWARD: Reduce research Waste And Reward Diligence
RIAS: responsible indicator for assessing scientists
SNIP: Source Normalized Impact per Paper
SWAN: Scientific Women's Academic Network
TOP: transparency and openness promotion
UMC: Utrecht Medical Centre
