# Supplementary material for: Assessing scientists for hiring, promotion, and tenure
Source: PLoS Biol. 2018 Mar 29;16(3):e2004089. doi: 10.1371/journal.pbio.2004089 (PMC5892914; doi:10.1371/journal.pbio.2004089)
Supplement: S1 Table — (DOCX) [file pbio.2004089.s001.docx]

| **WORKSHOP ORGANIZERS** | | |
| --- | --- | --- |
| **Name** | **Portfolio** | **Affiliation** |
| Steven Goodman, MD, MHS, PhD | Associate Dean of Clinical and Translational Research, Co-director of Meta-Research Innovation Center at Stanford and Professor of Medicine and of Health Research & Policy. | Stanford University |
| Frank Miedema, PhD | Dean and vice-chairman of the Executive Board of University Medical Center Utrecht, The Netherlands and professor of immunology. | University Medical Centre Utrecht, The Nethrelands |
| David Moher, PhD | Senior scientist, Clinical Epidemiology Program, Ottawa Hospital Research Institute, and Associate Professor, School of Epidemiology, Public Health and Preventive Medicine, Faculty of Medicine, University of Ottawa. | Ottawa Hospital Research Institute |
|  | | |
| **MEETING PARTICIPANTS** | | |
| **Name** | **Portfolio** | **Affiliation** |
| Jeremy M. Berg, PhD  Unable to participate | Editor-in-Chief of Science and the Science family of journals. | Science |
| Alastair Buchan, MD, PhD | Dean of Medicine and Head of the Medical Sciences Division at the University of Oxford. | University of Oxford |
| Stuart Buck, JD, PhD | Vice President of Research Integrity, Laura and John Arnold Foundation. | Laura and John Arnold Foundation |
| Ulrich Dirnagl, MD, PhD | Ulrich Dirnagl is director of the Department of Experimental Neurology, chief executive director of the Center for Stroke Research Berlin. | Centre for Stroke Research,  Berlin, Germany |
| Debbie Drake Dunne | METRICS founding executive director. | Stanford University |
| Daniele Fanelli, PhD | Senior Research Scientist for Meta-Research Innovation Center at Stanford. | Stanford University |
| Trish Groves (MBBS, MRCPsych) | Director of academic outreach, BMJ; honorary deputy editor, The BMJ; and editor-in-chief of BMJ Open. | BMJ |
| John P.A. Ioannidis, MD | C.F. Rehnborg Professor in Disease Prevention, and Professor of Medicine, of Health Research and Policy, Statistics, and co-Director, Meta-Research Innovation Center at Stanford. | Stanford University |
| Chonnettia Jones, PhD | Head of Insight and Analysis at Wellcome, an independent global charitable foundation dedicated to improving health through research, policy and engagement with society. | Wellcome Trust |
| Michael Lauer, MD | The Deputy Director for Extramural Research at the National Institutes of Health (NIH) | National Institute of Health |
| Malcolm Macleod, MD, PhD | Professor in Neurology and Translational Neuroscience at the Centre for Clinical Brain Sciences, University of Edinburgh, and Consultant Neurologist at NHS Forth Valley. | Centre for Clinical Brain Sciences, University of Edinburgh |
| Marcia McNutt, PhD | President of the National Academy of Sciences. | National Academy of Sciences |
| Sally C. Morton, PhD | Dean of the College of Science and Professor of Statistics at Virginia Tech. | Virginia Tech |
| Norman Rosenblum, PhD | Professor, Associate Dean, Physician Scientist Training, Depts. of Pediatrics, Physiology, Laboratory Medicine & Pathobiology, University of Toronto. Tier I Canada Research Chair in Developmental Nephrology. | University of Toronto |
| Daniel Sarewitz, PhD | Professor of Science and Society, and co-director and co-founder of the Consortium for Science, Policy, and Outcomes (CSPO; www.cspo.org), at Arizona State University. | Arizona State University |
| Rene von Schomberg, PhD | Agricultural scientist and STS specialist. | European Commission, |
| Paula Stephan, PhD | Professor of Economics, Andrew Young School of Policy Studies, Georgia State University. | Georgia State University |
| James Wilsdon, PhD | Professor of Research Policy and Director of Impact and Engagement in the Faculty of Social Sciences at the University of Sheffield (UK). | University of Sheffield (UK) |
| Paul Wouters | Professor of scientometrics and director of the Centre for Science and Technology Studies at Leiden University. | Leiden University, The Netherlands |
| Deborah A. Zarin, MD | Director of ClinicalTrials.gov. | ClinicalTrials.gov |
